# Botulinum Neurotoxin Devoid of Receptor Binding Domain Translocates Active Protease

**DOI:** 10.1371/journal.ppat.1000245

**Published:** 2008-12-19

**Authors:** Audrey Fischer, Darren J. Mushrush, D. Borden Lacy, Mauricio Montal

**Affiliations:** 1 Section of Neurobiology, Division of Biological Sciences, University of California San Diego, La Jolla, California, United States of America; 2 Departments of Biochemistry, Microbiology and Immunology, and Center for Structural Biology, Vanderbilt University Medical Center, Nashville, Tennessee, United States of America; Pasteur Institute, France

## Abstract

*Clostridium botulinum* neurotoxin (BoNT) causes flaccid paralysis by disabling synaptic exocytosis. Intoxication requires the tri-modular protein to undergo conformational changes in response to pH and redox gradients across endosomes, leading to the formation of a protein-conducting channel. The ∼50 kDa light chain (LC) protease is translocated into the cytosol by the ∼100 kDa heavy chain (HC), which consists of two modules: the N-terminal translocation domain (TD) and the C-terminal Receptor Binding Domain (RBD). Here we exploited the BoNT modular design to identify the minimal requirements for channel activity and LC translocation in neurons. Using the combined detection of substrate proteolysis and single-channel currents, we showed that a di-modular protein consisting only of LC and TD was sufficient to translocate active protease into the cytosol of target cells. The RBD is dispensable for cell entry, channel activity, or LC translocation; however, it determined a pH threshold for channel formation. These findings indicate that, in addition to its individual functions, each module acts as a chaperone for the others, working in concert to achieve productive intoxication.

## Author Summary

Botulinum neurotoxin, widely acknowledged to be the most potent toxin known, is a modular nanomachine and a marvel of protein design. This neurotoxin exploits a modular design to achieve its potent toxicity, which relies on one of its modules—the heavy chain channel—to operate as a specific protein translocating transmembrane chaperone for another of its component modules—the light chain protease. Our study shows that a di-modular protein consisting only of protease and translocation domains is sufficient to translocate active cargo into the cytosol of target cells. The receptor binding domain is dispensable for channel activity or LC translocation; however, it regulates the pH threshold of channel insertion into the membrane. The botulinum neurotoxin modular design embodies a tool for biomolecule delivery to predetermined target cells.

## Introduction

Botulinum neurotoxin (BoNT) inhibits synaptic exocytosis in peripheral cholinergic synapses causing botulism, a severe disease characterized by descending flaccid paralysis. *Clostridium botulinum* strains express seven BoNT isoforms known as serotypes A to G [1]. Each BoNT isoform is synthesized as a single polypeptide chain with a molecular mass of ∼150 kDa. The precursor protein is cleaved either by clostridial or host cell proteases into two polypeptide chains linked by a disulfide bridge [1]–[3]. Structurally, the mature toxin consists of three modules [1], [4]–[6]: a 50 kDa light chain (LC) Zn^2+^-metalloprotease, and the 100 kDa heavy chain (HC) which encompasses the N-terminal ∼50 kDa translocation domain (TD), also denoted H_N_ and the C-terminal ∼50 kDa receptor-binding domain (RBD) also termed H_C_.

This tri-modular architecture has a physiological counterpart. The RBD determines the cellular specificity mediated by the high affinity interaction with a surface protein receptor, SV2 for BoNT/A [7],[8] and BoNT/E [9], and synaptotagmins I and II for BoNT/B and BoNT/G [10], and a ganglioside (GT_1B_) co-receptor [7],[8],[10],[11]. Then, BoNTs enter sensitive cells via receptor-mediated endocytosis [1],[12],[13]. Exposure of the BoNT-receptor complex to the acidic milieu of endosomes [12]–[16] induces a conformational change leading to the insertion of the HC into the endosomal bilayer membrane, thereby forming transmembrane channels [17]–[20].

Previous studies have provided compelling evidence for the retrieval of a folded LC protease capable of proteolyzing its SNARE (soluble NSF attachment protein receptor) substrates, which are essential for synaptic vesicle fusion and neurotransmitter release [1],[2],[21],[22], only after productive translocation across lipid bilayer membranes and release from the channel [23]. These results indicate that the HC of BoNT/A acts as both a channel and a transmembrane chaperone for the LC to ensure a translocation competent conformation during LC transit [19],[23]. They also support the view that the TD module is the conduit for the passage of the LC module from the interior of the acidic endosome into the cytosol allowing access to the intracellular SNARE substrates [1],[13],[19],[23]. Thus, BoNT represents a fascinating example of molecular partnership: the HC chaperone activity driven by a pH gradient across the endosome prevents aggregation of the LC in the acidic vesicle interior, maintains the LC unfolded conformation during translocation, and releases the LC after it refolds at the neutral cytosolic pH. In the process, the HC channel is occluded by the LC during transit and open after completion of translocation and release of cargo [23]–[25].

Here, we investigated the minimal domain requirements for BoNT channel activity and for productive LC translocation. We characterized the isolated TD and the TD-disulfide linked to the catalytic LC using a translocation assay with single molecule resolution on excised membrane patches of neuroblastoma Neuro 2A cells [24],[25]. The system allowed us to dissect the minimal domain requirements and pH constraints for channel activity and for LC translocation. We utilized a cell-based neurotoxicity assay to assess the impact of the single molecule studies at the cellular level. The RBD was determined to be dispensable for both channel activity and LC translocation under mild acidic conditions (pH∼6). In contrast, the RBD restricted insertion of the TD into the membrane until localized to an acidic endosomal compartment where low pH (pH∼5) induced channel insertion concurrent with partial unfolding of the LC cargo, thereby triggering productive LC translocation and ultimate release into the cytosolic compartment.

## Materials and Methods

### Materials

Unless otherwise specified, all chemicals were purchased from Sigma-Aldrich. Purified native BoNT/A holotoxin and HC were from Metabiologics.

### Bacterial strains

*E. coli* strain DH5α cells were used in the cloning procedures and *E. coli* strains BL21.DE3.pLysS and BL21.DE3 (RIL) were used for protein over expression.

### Construction of BoNT/A LC-TD

DNA corresponding to the BoNT/A catalytic and translocation domains was obtained from two separate starting vectors: BoNT/A LC (unpublished results; a wild-type BoNT/A catalytic domain sequence) and TD-SDmut [26]; a wild-type BoNT/A TD amino acid sequence with silent mutations to disrupt an internal Shine Dalgarno site). The catalytic domain was amplified with primers that introduced a 5′ NdeI site (GGAATTCCATATGCCATTTGTTAATAAAC) and 3′ Sac1 site (GGCGAGCTCGCTTATTGTATCCTTTATCTAATG) and ligated into a TOPO vector (Invitrogen, Carlsbad, CA). An internal SacI site was then removed by QUIK-Change (Stratagene, La Jolla, CA) mutagenesis using the primer CTCTGGCACACGAACTGATCCACGCTGGTC and its reverse complement.

The BoNT/A TD was amplified with primers that introduced a 5′ SacI site (GCCGAGCTCTGAACGATCTGTGTATCAAAGTTAATAATTGGG) and 3′ XhoI site (CCGCTCGAGGTTCTTAATATATTCAGTAAATGTAG) and ligated into a TOPO vector. The catalytic and translocation domains were excised from the TOPO vectors using NdeI/SacI and SacI/XhoI, respectively and ligated into a pET24b vector that had been digested with NdeI/XhoI. The use of the engineered SacI site results in the insertion of an Arg between K447 (cat) and A448 (trans), a feature that was included in the design to improve the efficiency of trypsin activation.

### Expression and purification of the TD and LC-TD

The BoNT/A TD was expressed and purified from the TD-SDmut construct as described previously [26] with the following modification. BoNT/A TD was eluted from the Ni-affinity column in the presence of 0.2% dodecyl maltoside (DDM) and concentrated to 0.375 mg/ml in 0.2% DDM, 20 mM Tris, 150 mM NaCl, pH 8.0. The large dilutions used for single molecule studies insure that the effect of detergent on cell membranes is negligible. No detergent was required for LC-TD purification. BoNT/A LC-TD was expressed in *E.coli* BL21.DE3 (RIL) cells. A 10 ml aliquot was added to one L of Terrific Broth and induced at an OD = 600 nm using 1 mM isopropyl-β-D-thiogalactopyranoside (IPTG). Cells were harvested after overnight expression at 18°C and lysed using a French Press. The protein was purified on a Ni-NTA column followed by a Fast Flow Q Sepharose column and S200 gel filtration column. Di-chain BoNT/A LC-TD was generated by cleavage with trypsin: BoNT/A LC-TD (0.5 mg/ml) was incubated with 1 µg/ml trypsin overnight at 22±2°C. Thereafter, trypsin was inactivated with 0.25 mg/ml trypsin soybean inhibitor for 15 min at 22±2°C.

### Protein analysis

The purity of all BoNT/A proteins was determined qualitatively with SDS-PAGE analysis (Figure S1).

### Protease activity of BoNT/A proteins

Recombinant SNAP-25 [27],[28] was incubated with 60 ng of BoNT/A holotoxin or LC-TD in 13.2 mM Hepes (pH 7.4), 20 mM dithiothreitol, and 1 µM Zn (CH_3_COOH)_2_ for 30 min at 37°C. SDS-PAGE (12%) was used to visualize cleavage of SNAP-25 by BoNT/A proteins [29] (Figure S2).

### Cell culture and patch clamp recordings

Excised patches from Neuro 2A cells in the inside-out configuration were used as described [24],[25]. Current recordings were obtained under voltage clamp conditions. Records were acquired at a sampling frequency of 20 kHz and filtered online to 2 kHz with Gaussian filter. All experiments were conducted at 22±2°C.

### Solutions for patch clamp recordings

To emulate endosomal conditions the *trans* compartment (bath) solution contained (in mM) NaCl 200, NaMOPS [3-(N-morpholino) propanesulfonic acid] 5, (pH 7.0 with HCl), tris-(2-carboxyethyl) phosphine (TCEP) 0.25, ZnCl_2_ 1, and the *cis* compartment (pipet) solution contained (in mM) NaCl 200, NaMES [2-(N-morpholino) ethanesulfonic acid] 5, (pH 5.3 or pH 6.0 with HCl). When the *cis* compartment was tested with pH 7 buffer, the *trans* compartment solution set to pH 7.0 was used. The osmolarity of both solutions was determined to be ∼390 mOsm. ZnCl_2_ was used to block endogenous channel activity specific to Neuro 2A cells [30],[31]. BoNT protein reconstitution and channel insertion was achieved by supplementing 2.5–5 µg/ml BoNT holotoxin, HC, LC-TD, TD, to the pipet solution, which was set to pH 5.3, pH 6.0 or pH 7.0.

### Single-channel data analysis

Analysis was performed on single bursts of each experimental record. Only single bursts were analyzed due to the random duration of quiescent periods. A single burst is defined as a set of openings and closings lasting ≥50 ms bounded by quiescent periods of ≥50 ms before and after. Single-channel conductance (γ) was calculated from Gaussian fits to current amplitude histograms. The total number of opening events (*N*) analyzed was 165,936. Time course of single-channel conductance change for each experiment was calculated from γ of each record, where t = 0 s corresponds to onset of channel activity, and average time course was constructed from the set of individual experiments for a single condition. The half-time for completion of single-channel conductance change event (*t*_½_) was calculated from Sigmoidal fit to the average time course. The voltage-dependence of channel opening was calculated from measurements of the fraction of time that the channel is open (*P_o_*) as a function of voltage by integration of γ histograms where γ is 60≤γ≤75 pS. Statistical values represent means±SEM, unless otherwise indicated. *n* denotes the number of different experiments.

### Cell-based intoxication assay

Cleavage of endogenous SNAP-25 within Neuro 2A cells exposed to BoNT/A and truncation proteins was investigated as described [32]; Neuro-2A cells were seeded at a density of ∼120,000 cells per well in a 12-well tissue culture plate in DMEM culture medium. After incubation for 24 h, the media were removed and replaced with serum-free media containing 5.0 µg of BoNT/A holotoxin, LC-TD or TD. After incubation for ∼48 h, the cells were harvested by removing the media, adding 160 µl of 1× NuPAGE SDS sample buffer (Invitrogen), and boiling for 10 min.

### Western blot analysis

SDS-PAGE and Western blotting were conducted using standard protocols. Proteins within the whole-cell extract samples were separated by SDS-PAGE on a 12% Bis-Tris NuPAGE gel in MOPS/SDS running buffer (Invitrogen) before transfer to a 0.2 µm nitrocellulose membrane for 120 min at 30°C [29]. After blocking in 2% skim milk/H_2_O for 20 min at room temperature, the membrane was washed three times for 5 min at room temperature with TBST [25 mM Tris (pH 7.4), 137 mM NaCl, 2.7 mM KCl, and 0.1% Tween 20]. Primary antibody, anti-SNAP-25 mouse monoclonal IgG_1_ (200 µg/ml; Santa Cruz Biotechnology, Santa Cruz, CA) diluted 1∶1,000 into 2% skim milk/H_2_O, was added, and the blot was incubated for 20 min at room temperature followed by four 5-min washes with TBST at room temperature. Next, secondary antibody, goat anti-mouse HRP-conjugated (10 µg/ml; Pierce, Rockford, IL) diluted 1∶500 into 2% skim milk/H_2_O, was added, and the blot was incubated for 1 h at room temperature followed by washing for 120 min at room temperature. Bands were visualized with 4 ml of SuperSignal West Dura Chemiluminescent Substrate (Pierce) and analyzed with an X-OMAT 2000A processor (Kodak). Western blot analysis was quantified with the use of Image J software (NIH).

## Results

### The channel activity of BoNT/A is confined to the TD

**Figure 1:**
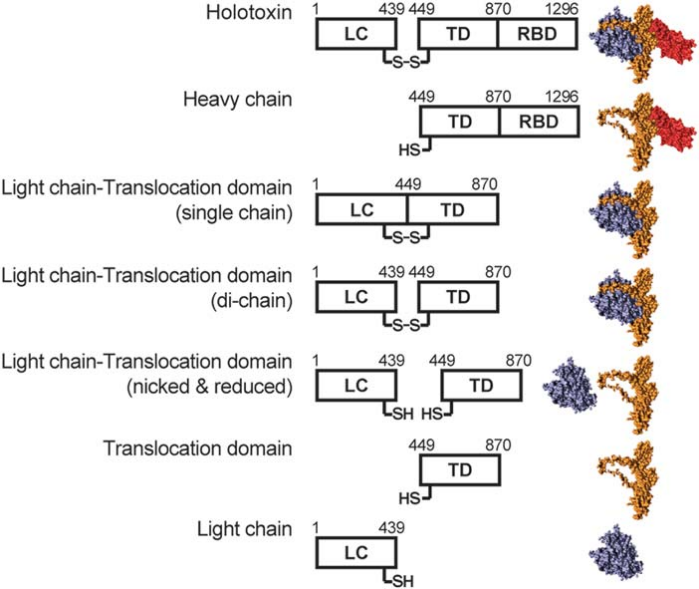
Modular organization of BoNT/A holotoxin and the different constructs studied. (Top Panel) Schematic representation of BoNT/A holotoxin showing domain organization; LC denotes light chain, TD, translocation domain, and RBD, receptor binding domain. Numbers indicate amino acid number from the sequence of BoNT/A and represent the boundaries between individual modules. The disulfide bond between the LC and the HC in the mature toxin is indicated by −S-S−. The same convention applies for the proteins represented in the second to seventh panels: the di-modular heavy chain, HC; the single-chain di-modular light chain-translocation domain, LC-TD; the di-chain di-modular LC-TD; the LC-TD after nicking and reduction (indicated by −SH HS−), the isolated, single module translocation domain, TD, and the isolated single module LC. The structure of BoNT/A holotoxin (PDB accession code 3BTA [4]) and models of the different constructs derived from the holotoxin structure are displayed adjacent to the corresponding protein. The solvent accessible surfaces were rendered using YASARA (www.yasara.org); the LC is represented in purple, the TD in orange, and the RBD in red.

Earlier studies were suggestive of channel forming activity by the N-terminal half of the HC (H_N_) [17]. We generated several truncation constructs of BoNT/A holotoxin, purified them and examined their channel activity; these include the HC, H_N_ − the N-terminal half of the HC called the translocation domain (TD) [26], and the LC and TD linked by a disulfide bridge (LC-TD) [33] (Figure 1). The LC-TD was expressed as a single polypeptide chain of ∼100 kDa with a disulfide bridge; the functionally relevant di-chain protein was generated by trypsin cleavage of the linker between the disulfide bridge cysteine residues. Trypsin nicking did not disrupt the disulfide crosslink, as demonstrated by SDS PAGE analysis (Figure S1), or affect the enzymatic activity of the LC (Figure S2).

**Figure 2:**
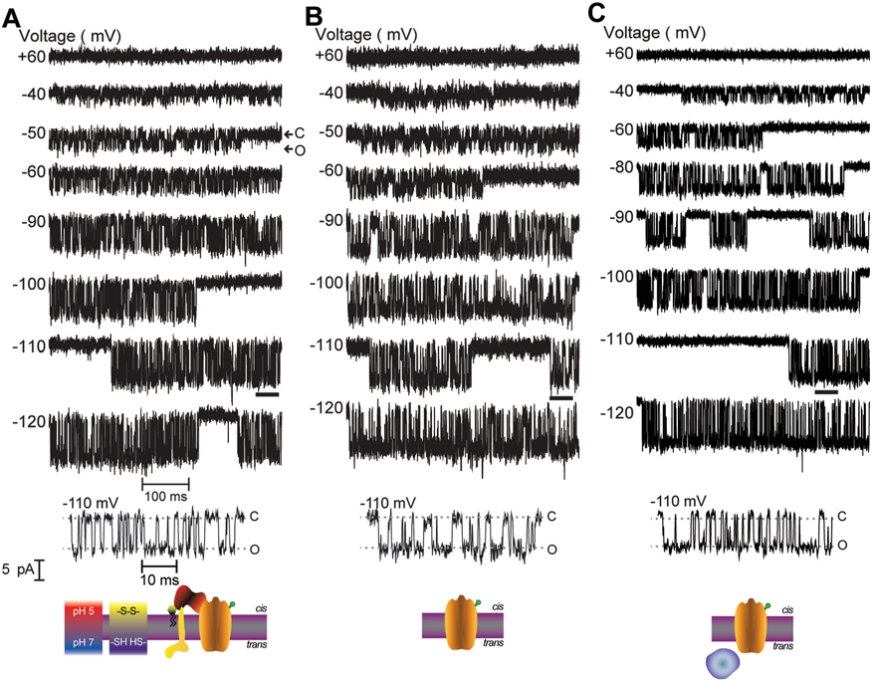
BoNT/A HC, TD, and LC-TD channel activity measured on excised patches of Neuro 2A cells. Representative single-channel currents at the indicated voltages; consecutive voltage pulses applied to the same patch for each experimental condition. Channel opening is indicated by a downward deflection; C and O denote the closed and open states. γ values for HC (A) = 65.3±0.4 pS, TD (B) = 64.4±0.4 pS, and LC-TD (C) = 69.2±0.9 pS. The sections of the recordings obtained at −110 mV delimited by the black bars are shown in the bottom traces at a 10-fold higher time resolution; the prototypical square events which are characteristic of unitary channel currents are clearly discerned. An interpretation of the results is schematically illustrated at the bottom of each panel: The membrane is depicted as a grey bar with magenta boundaries; LC–purple, TD–orange, RBD–red, and the cysteine involved in the LC-HC disulfide crosslink–green sphere; the SV2 protein-receptor is illustrated in yellow and the GT1b co-receptor in black (ceramide moiety inserted in the bilayer) and lime (the polar head group); the pH and redox conditions on the *cis* and *trans* compartments are indicated. These conventions also hold for Figure 4.

Channel formation was monitored on excised membrane patches from neuroblastoma cells under conditions that recapitulate the pH and redox gradients across endosomes: the *cis* compartment, defined as the compartment containing BoNT/A proteins, was held at pH 5.3 and the *trans* compartment was maintained at pH 7.0 and supplemented with the membrane nonpermeable reductant tris-(2-carboxyethyl) phosphine (TCEP). The LC-TD (Figure 2C), and TD (Figure 2B) exhibited channel activity with characteristics indistinguishable from those of BoNT/A HC (Figure 2A): channel activity occurred in bursts of fast transitions between closed and open states interspersed between periods of no channel activity; discrete channel openings displayed a distinctive single-channel conductance (γ)∼65 pS (in symmetric 0.2 M NaCl), with a high probability to reside in the open state (*P_o_*), as illustrated by the records depicted in the corresponding bottom traces displayed at higher time resolution. The LC-TD and TD display a voltage dependence similar to the unoccluded BoNT channel which resulted after completion of LC translocation [24],[25]; V_1/2_, the voltage at which *P_o_* = 0.5, −67.2±2.9 mV for holotoxin, −59.0±9.1 mV for LC-TD, and −64.0±4.2 mV for TD. Together, these results demonstrate that the channel activity of BoNT is confined to the TD.

### BoNT/A TD forms channels at neutral pH and in the absence of a transmembrane pH gradient

**Figure 3:**
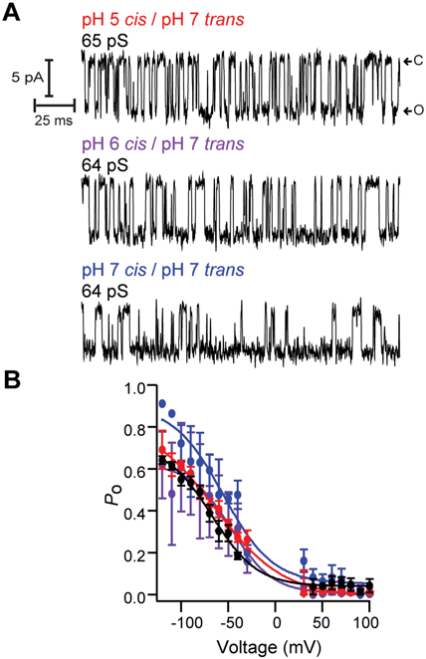
BoNT/A TD channel activity is independent of a transmembrane ΔpH. (A) Top panel illustrates channel activity monitored with ΔpH 5 *cis*/7 *trans*, middle panel shows ΔpH 6 *cis*/7 *trans*, and bottom panel shows no ΔpH, 7 *cis*/7 *trans*. Representative single-channel currents recorded at −100 mV. After GΩ seal formation, channel activity begins at 30 min (top), 14 min (middle), and 30 min (bottom). Low conductance intermediates were not observed. (B) Analysis of unoccluded channel activity for BoNT/A holotoxin for pH 5/pH 7 (black circle) (V_½_ = −67.2±2.9 mV), BoNT/A TD for pH 5/pH 7 (red circle) (V_½_ = −64.0±4.2 mV), pH 6/pH 7 (magenta circle) (V_½_ = −46.8±3.1 mV), pH 7/pH 7 (blue circle) (V_½_ = −55.3±3.8 mV).

We previously demonstrated that channel activity for BoNT HC depends on the presence of a pH gradient (ΔpH) across the membrane [23]–[25]. The HC is a di-modular protein consisting of the TD and the RBD (Figure 1). Given that the TD forms channels similar to those of the HC (Figure 2), the question arises as to whether the RBD modulates the TD channel activity, particularly with regards to the observed dependence on ΔpH. As shown in Figure 3A, the TD channel activity was practically equivalent when the *cis* compartment solution was adjusted to pH 6 or pH 7, thereby reducing (top and middle records) or eliminating (bottom record) the ΔpH. Channel activity retained the hallmark features of the channel measured for the isolated HC [23],[34] and for holotoxin after completion of productive translocation [24],[25] irrespective of ΔpH, namely, the bursting pattern of channel activity with an invariant γ at ∼65 pS (Figure 3A). The voltage dependence of the *P_o_* was modulated by attenuating or eliminating ΔpH: The V_1/2_ was right shifted to ∼−47 mV when the *cis* compartment was maintained at pH 6.0 for TD (Figure 3B). The interactions between the TD and the RBD therefore modulate the pH threshold for membrane insertion and channel formation; in the absence of the RBD the TD readily forms channels at neutral pH and in the absence of a ΔpH.

### BoNT/A LC-TD forms a protein-conducting channel that translocates LC in a pH-dependent manner

**Figure 4:**
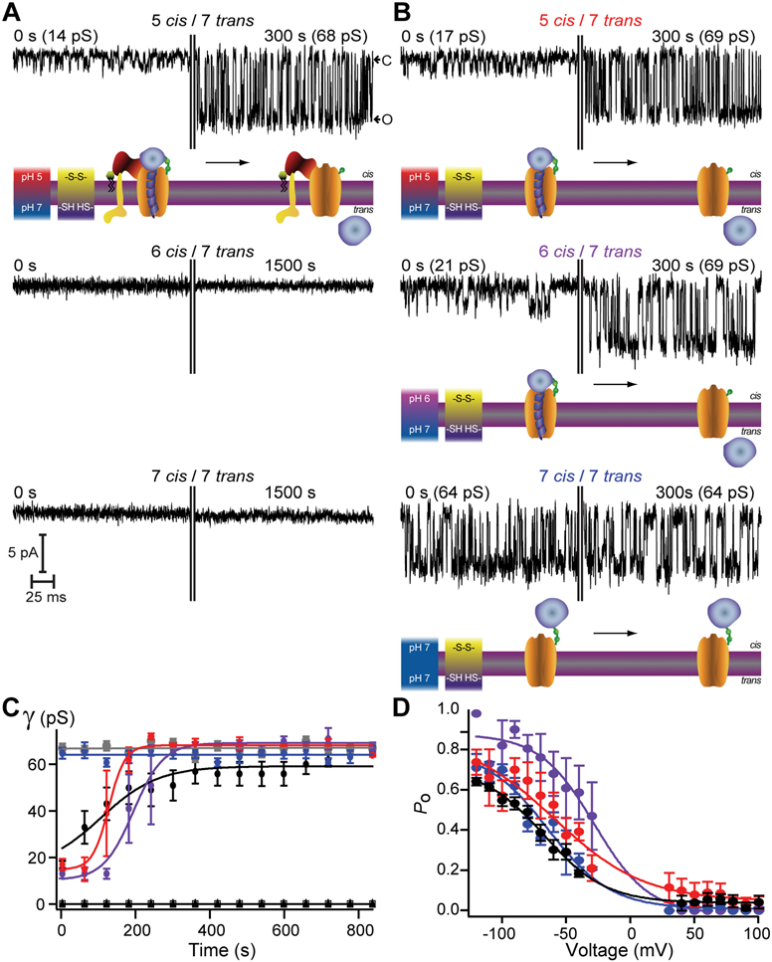
BoNT/A holotoxin and LC-TD channel activity in excised patches of Neuro 2A cells over a range of pH values in the cis compartment. (A and B) Top panels illustrate channel activity monitored with ΔpH 5 *cis*/7 *trans*, middle panels show ΔpH 6 *cis*/7 *trans*, and bottom panels show ΔpH, 7 *cis*/7 *trans*. (A) BoNT/A holotoxin channel activity elicited under pH 5/pH 7 begins 10 min after GΩ seal formation, t = 0 s, and transitions from a low conductance intermediate state to the unoccluded state after completion of LC translocation. The vertical lines indicate gaps to accommodate the full recording in the limited space. A schematic representation is depicted under the records for panels A and B. BoNT/A holotoxin does not form channels under pH 6/pH 7 or pH 7/pH 7. B, BoNT/A LC-TD channel activity begins 10 min, 12 min, and 45 min after GΩ seal formation for pH 5/pH 7, pH 6/pH 7, pH 7/pH 7, respectively. (C) Average time course of conductance change for BoNT/A holotoxin pH 5/pH 7 (black circle) (*t*_1/2_ = 105±20 s), HC pH 5/pH 7(gray square), LC-TD pH 5/pH 7 (red circle) (*t*_1/2_ = 130±10 s), LC-TD pH 6/pH 7 (magenta circle) (*t*_1/2_ = 190±10 s), and LC-TD pH 7/pH 7 (blue circle), (3≤*n*≤6 per data point; average *N* per data point = 829 events). No channel activity was detected for BoNT/A holotoxin under pH 6/pH 7 (black triangle) (*n* = 8) and pH 7/pH 7 (black square) (*n* = 8) conditions. (D) Analysis of unoccluded channel activity for BoNT/A holotoxin pH 5/pH 7 (black circle) (V_½_ = −67.2±2.9 mV), BoNT/A LC-TD for pH 5/pH 7 (red circle) (V_½_ = −59.0±9.1 mV), BoNT/A LC-TD for pH 6/pH 7 (magenta circle) (V_½_ = −28.1±4.5 mV), BoNT/A LC-TD for pH 7/pH 7 (blue circle) (V_½_ = −64.1±2.9 mV).

Translocation of BoNT/A LC by the BoNT/A channel can be monitored in real time and at the single molecule level in excised membrane patches from neuroblastoma cells [24],[25]. LC translocation through the HC channel requires conditions which emulate those prevalent across endosomes. Translocation was observed as a time-dependent increase in Na^+^ conductance through the HC channel recorded at −100 mV (Figure 4A, top panel). Initial channel activity exhibited small, discrete events with γ∼14 pS (Figure 4A, top, left panel). Progressively, γ underwent a continuous increase until reaching a stable value of 68 pS (Figure 4A, top, right panel), a constant conductance monitored for the duration of the experiment (Figure 4C, black circle). This steady-state γ was also the characteristic conductance of isolated HC recorded under identical conditions [24],[25],[34]. The half-time for completion of such an event (*t*_½_), estimated from the transition to high conductance, was ∼150 s (Figure 4C).

We interpret the intermediate conductance states as reporters of discrete transient steps during the translocation of the LC across the membrane; a schematic representation is depicted under the records shown in Figure 4A, top [24],[25]. During protease translocation, the protein-conducting channel progressively conducts more Na^+^ around the polypeptide chain before entering an exclusively ion-conductive state. For BoNT holotoxin, channel formation and LC translocation are dependent on ΔpH; no channels were detected when the internal solution containing BoNT was held at pH 6 (Figure 4A, middle trace) or pH 7 (Figure 4A, bottom trace) (Figure 4C, black triangle and black square), in agreement with our previous findings [23]. In contrast, LC-TD translocation (Figure 4B, top panel) proceeded even under a modest ΔpH (6 on the *cis*- and 7 on the *trans*- compartments) (middle trace). Remarkably, when the excised membrane patches were bathed in symmetric neutral pH solutions, the LC-TD formed HC-like channels; low conductance intermediate states were not detected (Figure 4B, lower panel, and 4C, blue). Circular dichroism analysis of the LC at pH 7 indicates a high α-helical content incompatible with a translocation competent conformation [23]. These results imply that the LC remains folded at pH 7 and therefore cannot go through the ∼15 Å diameter of the TD channel [23],[35], thereby allowing expression of the TD channel activity unperturbed by cargo.

### BoNT/A LC-TD cleaves its substrate, SNAP-25, in intact Neuro 2A cells

**Figure 5:**
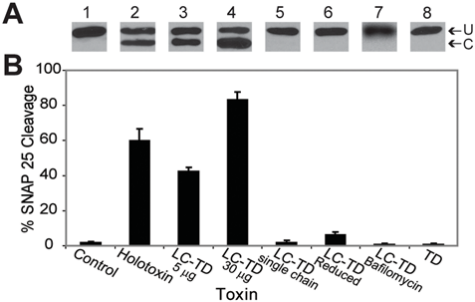
BoNT/A cleavage of endogenous SNAP-25 within Neuro 2A cells. (A) Representative western blot and (B) extent of SNAP-25 cleavage (average %) measured from western blots. (A) U and C denote uncleaved and cleaved SNAP-25. Blot shows SNAP-25 in the absence of BoNT/A - control (lane 1), SNAP-25 cleaved with 5 µg/well BoNT/A holotoxin (lane 2), with 5 µg/well (lane 3) and 30 µg/well (lane 4) LC-TD. SNAP-25 was unaffected by incubation of cells with 5 µg/well BoNT/A LC-TD single chain (lane 5) or reduced with TCEP prior to incubation with cells (lane 6), by incubation with 2 µM bafilomycin and 5 µg/well BoNT/A LC-TD (lane 7) and by 5 µg/well BoNT/A TD (lane 8). (3≤*n*≤per data point).

The findings shown in Figure 4 imply that, while BoNT/A holotoxin readily enters neuronal cells via receptor-mediated endocytosis, the LC-TD devoid of the RBD would insert and be trapped in the plasma membrane unable to access its cytosolic substrate SNARE protein. To test this notion, we utilized a cell-based assay that monitors the amount of intact versus cleaved endogenous SNAP-25 protein within Neuro-2A cells [32]. This assay is highly reliable when given a 48 hr exposure to the BoNT proteins. In the absence of BoNT/A holotoxin or in the presence of isolated TD [26], SNAP-25 remained intact (Figure 5A, lanes 1 and 8), whereas in the presence of BoNT/A holotoxin, a lower-molecular-weight proteolysis product of ∼24 KDa was detected (Figure 5A, lane 2). Incubation of cells with LC-TD resulted in proteolysis of SNAP-25 (Figures 5A and 5B, lanes 3 and 4). The extent of proteolysis attained by LC-TD was comparable to that produced by holotoxin, albeit it required higher protein concentration consistent with a lower efficacy. A single-chain BoNT/A LC-TD unexposed to trypsin (lane 5) or the LC-TD that had been trypsin nicked and the disulfide bridge reduced prior to the assay (lane 6) did not cleave SNAP-25. Remarkably, cleavage of SNAP-25 by BoNT/A LC-TD does not occur when cells are preincubated with 2 µM bafilomycin, an inhibitor of the vesicular proton pump and, therefore, of endosomal acidification [36]–[38] (lane 7). These results indicate that BoNT/A LC-TD enters neurons without the aid of the RBD. Translocation requires nicking of the LC cargo from the TD carrier and does not arise from leaky cells that uptake LC via a non-specific mechanism. The implication is, therefore, that the inserted LC-TD uses the constitutive endocytic pathway to enter cells, is subsequently processed and undergoes translocation of LC with the consequent cleavage of the intracellular substrate. Together, the results shown in Figures 4 and 5 provide compelling evidence that the RBD is not necessary for channel activity or LC translocation, and that the LC-TD is a di-modular BoNT endowed with the ability to deliver folded and active LC protease into the cytosol of target cells.

## Discussion

### The di-modular LC-TD protein embodies a minimal design for translocation of active cargo

The tri-modular holotoxin encompassing the LC protease, the TD and the RBD enters neurons *via* receptor mediated endocytosis determined by the RBD module, and achieves the cytosolic co-localization of the LC protease with its substrate by the chaperone activity of a TD protein-conducting channel. Here we demonstrate the BoNT channel-forming entity is confined to the TD (Figure 2) which, at variance with the holotoxin and the di-modular HC, displays channel activity irrespective of a transmembrane ΔpH (Figure 3). We further show that for the di-modular HC the pH threshold for membrane insertion and channel formation is modulated by the interactions between these two modules and/or by the RBD interaction with the SV2 receptor (Figure 4A and 4C). Previous studies indicate that channel formation occurs concomitantly with protein translocation; therefore we investigated the minimal domain requirements for productive LC translocation. We established that the di-modular LC-TD is sufficient for productive translocation of active protease (Figure 4 and Figure 5) given that the RBD responsible for cell binding and internalization was unnecessary for BoNT/A LC translocation (Figure 4B). These results are similar to those obtained for the translocation of diphtheria toxin catalytic domain by its translocation domain [39]. The absence of the RBD confers to the LC-TD a wider pH range for translocation activity (Figure 4). Having identified this minimal entity, the next set of questions is what this tells us about the BoNT tri-modular design in the context of cellular toxicity. The following inferences can be derived from our analysis.

### Role of the receptor binding domain in BoNT neurotoxicity

The RBD determines targeting of BoNT to the peripheral nervous system and insures efficient intoxication by at least three mechanisms. In foodborne botulism, the RBD binds receptors on the mucosal surface of gut epithelial cells independent of associated BoNT complex proteins [13],[40]. The holotoxin then undergoes receptor mediated endocytosis and transcytosis with subsequent delivery to the basolateral side of the epithelial cell [40]–[42]. Once released into the circulation, BoNT reaches cholinergic nerve terminals and a second round of cell entry occurs. BoNTs enter sensitive neurons *via* receptor-mediated endocytosis determined by its high affinity interaction with a surface protein receptor and a ganglioside co-receptor [1], [7], [8], [10]–[13]. During cell binding and intracellular traffic, the RBD restricts the TD from membrane insertion until its residence within the acidic interior environment of endosomes. Without the RBD, the TD readily inserts into the plasma membrane of neuronal cells and forms channels (Figure 3). For the LC-TD, the LC remains tethered to its TD carrier on the cell surface by the disulfide linkage, and folded in the extracellular neutral pH environment. Accordingly, the RBD serves to chaperone the LC and TD, insuring that partial unfolding of the LC is concomitant with TD channel formation thereby promoting productive LC translocation. A similar example of a toxin RBD-receptor interaction regulating the pH threshold required for pore formation has been observed for anthrax toxin [43] and likely reflects intoxication requirements shared between the two toxins. The lack of structural similarity between BoNT and anthrax toxin [44], however, suggests that the mechanisms by which this regulation occurs will be unique. A LC-RBD di-modular protein has not been characterized thus far. We conjecture that such an entity would bind to peripheral neurons and undergo receptor-mediated endocytosis; however, in the absence of concurrent cargo translocation, the LC protease would be irreversibly inactivated by permanent exposure to the acidic endosome interior.

### Paradox of LC-TD channel activity versus cellular specificity

At neutral pH, why does the LC-TD translocate from the plasma membrane in cell-based neurotoxicity assays but not in the single molecule studies? To understand this apparent discrepancy, we first consider the properties of the individual BoNT modules in the context of neuronal cells. The isolated LC, a globular protein with a thermolysin-like fold at neutral pH [4],[45], does not enter neurons to cleave its cytosolic substrate [1],[23]. Multiple studies have demonstrated that, upon exposure to acidic pH, the LC partially unfolds and loses catalytic activity, whereas it remains folded and enzymatically active at neutral pH [23],[46],[47]. The LC requires the TD for access to the cytosol, but the conductance of TD channels suggests a pore size of ≤15 Å in diameter [23],[25],[34],[35]. Therefore, the LC must enter an acidic environment to translocate into the cytosol. In the absence of RBD, the TD inserts into the membrane of Neuro 2A cells and would be open at the negative membrane potentials prevalent at the cell membrane (Figure 4B). Thus, the TD channel would dissipate the electrochemical gradients across the plasma membrane, potentially disrupting normal cellular function and activating both the constitutive and regulated endocytosis pathways to recover cellular homeostasis. By usurping the endocytotic machinery and entering acidic endosomes, translocation of the tethered LC could then proceed [23]–[25]. Completion of translocation and release of the LC requires the reduction of the disulfide linkage [23]–[25]. Consistent with this notion is the finding that cells exposed to either isolated LC or single chain LC-TD preserve intact their endogenous SNAP-25 content (Figure 5). Furthermore, and in accordance with our findings, is the documented ability of LC-TD to induce paralysis in mice at relatively high protein concentrations [33]. The fact that BoNT/A LC-TD intoxicates neurons in the absence of the neuronal targeting RBD implies that LC-TD may be developed into a molecular device with a broad spectrum of cellular specificity. An initial evaluation of the translocation activity of LC-TD in a number of non-neuronal cell lines, which thus far have included CHO (derived from Chinese hamster ovary cells) and Vero (derived from monkey kidney epithelial cells) cells, show that these cells do not display translocation activity. The difference between these cultured epithelial cells and neuronal cells is likely to be more complex than just the absence of a toxin receptor and co-receptor, as the endo/exocytotic machinery and the membrane lipid composition may vary in substantial and deterministic ways. This issue constitutes a new line of investigation which may provide unsuspected insights into pathways or components involved in neuronal trafficking or recycling of neurotransmitter vesicles.

### BoNT as a modular nanomachine

The findings presented here highlight the molecular design of BoNT, a modular machine in which each component functions individually yet their tight and concerted interplay implies that each domain serves as a chaperone for the others. The RBD insures that TD channel formation occurs concurrently with LC unfolding to a translocation competent conformation (Figure 4B and 4C). At the positive membrane potential prevailing across endosomes the TD channel would be closed until it is gated by the LC to initiate its translocation into the cytosol [34]. The TD itself protects the LC within the acidic milieu of the endosome interior, chaperones the LC to the cytosol, and releases the LC in an enzymatically active conformation to access its cytosolic substrate SNARE proteins [23]. This modular design of BoNT and similar modular toxins [44] is therefore emerging as a tool for biomolecule delivery to predetermined target cells [38], [48]–[54]. Model cargo proteins have been tethered to enzymatically inactive BoNT and demonstrated to translocate and function within the neuronal cell [51]. These studies have focused on inactivation or removal of the enzymatic domain and tethering of additional cargo proteins. By contrast, replacement of the neuronal targeting RBD with one that recognizes a distinct, unique cellular surface protein (for reviews see [2], [44], [52]–[54]) could transform BoNT TD into a widespread delivery system for a diverse array of cargo proteins to the target tissue of choice, provided cargo proteins reversibly unfold and refold at the beginning and the end of translocation thereby retaining a tight association with the protein-conducting channel throughout the process.

## Supporting Information

Figure S1 Coomassie blue stained SDS-PAGE analysis of BoNT/A holotoxin, LC-TD, and TD; numbers denote *Mr* Standards in kDa; DTT (+) (−) indicate presence or absence of 100 mM dithiothreitol in the sample loading buffer.
(1.23 MB PDF)

Figure S2 Coomassie blue stained SDS-PAGE analysis of *in vitro* cleavage of SNAP-25 by BoNT/A holotoxin and LC-TD. Lane 1 shows SNAP-25 in the absence of BoNT/A - control; lane 2, a 21 kDa molecular weight standard protein; lanes 3 and 4 show SNAP-25 cleaved by BoNT/A holotoxin and by LC-TD. Both BoNT/A holotoxin and LC-TD cleaved SNAP-25 to completion as compared to the uncleaved control presented in lane 1.
(1.32 MB PDF)
