# Supplementary figures and images for: Botulinum Neurotoxin Devoid of Receptor Binding Domain Translocates Active Protease
Source: PLoS Pathog. 2008 Dec 19;4(12):e1000245. doi: 10.1371/journal.ppat.1000245 (PMC2596314; doi:10.1371/journal.ppat.1000245)

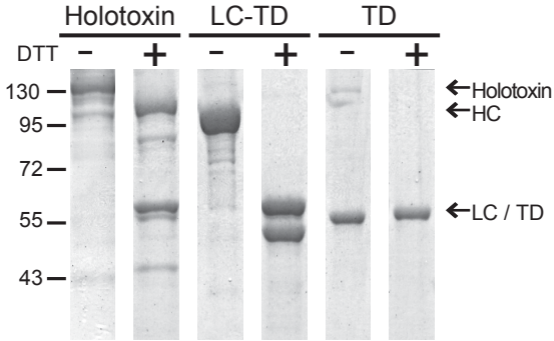

Supplement: Figure S1 — Coomassie blue stained SDS-PAGE analysis of BoNT/A holotoxin, LC-TD, and TD; numbers denote Mr Standards in kDa; DTT (+) (−) indicate presence or absence of 100 mM dithiothreitol in the sample loading buffer. (1.23 MB PDF) [file ppat.1000245.s001.pdf]

1

2

3

4

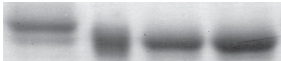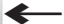

SNAP-25

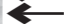

Cleaved SNAP-25

Supplement: Figure S2 — Coomassie blue stained SDS-PAGE analysis of in vitro cleavage of SNAP-25 by BoNT/A holotoxin and LC-TD. Lane 1 shows SNAP-25 in the absence of BoNT/A - control; lane 2, a 21 kDa molecular weight standard protein; lanes 3 and 4 show SNAP-25 cleaved by BoNT/A holotoxin and by LC-TD. Both BoNT/A holotoxin and LC-TD cleaved SNAP-25 to completion as compared to the uncleaved control presented in lane 1. (1.32 MB PDF) [file ppat.1000245.s002.pdf]
